# Exploring the Influence of Genetic Single-Nucleotide Polymorphism (SNPs) on Endodontic Pathologies: A Comprehensive Review

**DOI:** 10.7759/cureus.74389

**Published:** 2024-11-25

**Authors:** Ali M Falatah, Salihah A Alturki, Abdulatif I Aldahami, Nourh A Alrashidi, Yahya Sinnah, Rowaida M Aldgeel, Khalid Z Alanazi, Amira S Alkhaled, Talal S ALjuaid, Naif H Alotaibi, Worod J Altwijri

**Affiliations:** 1 Endodontics, King Fahad Hospital, Madinah, SAU; 2 General Dentistry, Ministry of Health, Al-Qassim, SAU; 3 Dentistry, Qassim University, Bachelor Degree of Dental Surgery (BDS), Buraydah, SAU; 4 College of Dentistry, Mustaqbal University, Buraydah, SAU; 5 Dentistry, King Khalid University, Abha, SAU; 6 General Dentistry, Ministry of Health, Jeddah, SAU; 7 Dentistry, Alexandria University, Hafar Al Batin, SAU; 8 College of Dentistry, King Saud bin Abdulaziz University for Health Sciences, Riyadh, SAU; 9 College of Dentistry, Taif University, Taif, SAU; 10 College of Dentistry, King Saud University, Riyadh, SAU; 11 Dentistry, Princess Nourah Bint Abdulrahman University, Riyadh, SAU

**Keywords:** endodontics, genetic polymorphisms, inflammatory response, pulpal and periapical diseases, single nucleotide polymorphisms (snps)

## Abstract

A considerable portion of the global population is affected by pulpitis and periapical lesions. While the impact of infections caused by various microbes and host effector molecules in pulpal and periapical diseases is widely recognized, disease susceptibility and progression are also influenced by the dynamic interaction between host genetic factors and environmental influences. Apical periodontitis occurs as an inflammatory response to microorganisms present in the root canals of infected teeth. Initially functioning as the body’s defense mechanism, this response often progresses to chronic inflammation. Several studies have established associations between genetic polymorphisms and various dental conditions, including temporomandibular joint (TMJ) disorders, dental caries, orthognathic surgeries, open bite malocclusion, periapical periodontitis, pulp stones, pulpitis, periapical abscesses, local anesthesia complications, and endodontic treatment outcomes. Key findings from this review highlight the role of specific single-nucleotide polymorphisms (SNPs) in genes such as matrix metalloproteinase (MMP)1, MMP2, MMP3, interleukin (IL)-1β, IL-6, IL-17, and tumor necrosis factor-alpha (TNF-α), which influence inflammatory pathways and tissue remodeling. For example, SNPs in interleukin genes, such as IL-1β (-511 C/T), have been linked to an increased risk of apical periodontitis, while MMP gene polymorphisms contribute to tissue degradation in periapical lesions.

This review underscores the importance of identifying genetic markers that drive disease progression and inflammatory processes in pulpal and periapical pathologies. A better understanding of these mechanisms can inform strategies for disease prevention, personalized treatment approaches, and improved endodontic outcomes.

## Introduction and background

A significant portion of the global population suffers from pulpal and periapical pathologies. Its prevalence is largely attributed to the widespread presence of primary causative factors such as dental caries and traumatic injuries. Dental caries remains one of the most common diseases affecting the human population, particularly among younger age groups. There is a proven relationship between dental caries and inflammation in the dental pulp tissue, even during the early stages. If caries is diagnosed early, it can be treated with restorative procedures. However, since dental caries are often asymptomatic, many cases progress to irreversible pulpitis [1]. Studies estimate that approximately 5% to 25% of the global population may experience apical periodontitis at some point in their lives, with a systematic review and meta-analysis reporting that 52% of adults worldwide have at least one tooth with apical periodontitis and that the prevalence at the tooth level is approximately 5% [1-7]. Apical periodontitis then develops as an inflammatory response. While it acts as the body's defense mechanism against microorganisms present in the root canals of infected teeth, it leads to chronic inflammation. Individual genetic factors can also influence its etiology, as demonstrated by specific genetic variations associated with susceptibility to periapical pathologies [2].

Furthermore, persistent infections caused by various microorganisms within the root canal system typically lead to the necrosis of pulpal tissues and the development of apical periodontitis. These microorganisms and their virulence factors trigger the host immune system, recruiting different cell types and mediators. The resulting immune response causes the degradation of supporting tissues and the formation of periradicular lesions [3].

In patients with pulpitis, the possibility of conservative management, which aims to maintain the vitality of the dental pulp, depends on the condition of the pulp. If the affected tooth’s pulp is vital and healthy, it can be restored and preserved through vital pulp therapy [1]. However, if the dental pulp is necrotic, root canal therapy becomes necessary. Successful root canal therapy relies on proper instrumentation, thorough disinfection, and adequate obturation. In addition, healing of the periapical tissues is essential to achieve favorable outcomes. Nevertheless, post-treatment pain remains a common complication that affects 3% to 58% of patients [2]. Successful root canal therapy can also resolve apical periodontitis, but complete sterilization of an infected root canal is not always achievable, and residual lesions may persist [4]. Reviews suggest a potential correlation between apical periodontitis and systemic health issues. Therefore, to mitigate its impact on general health, it is crucial to understand the factors that drive apical periodontitis and prevent its onset [5,6].

Current research suggests that disease progression and treatment outcomes in apical periodontitis are influenced by the complex interaction between microbial factors and genetic factors. By affecting inflammatory and immune responses in different ways, differences in genetic makeup lead to varying reactions to environmental factors [1,8]. This review aims to explore recent discoveries regarding the genetic factors and polymorphisms influencing pulpal and periapical reactions that lead to irreversible pathologies. Numerous studies have investigated single-nucleotide polymorphisms (SNPs) in various genes - such as matrix metalloproteinases (MMPs) and their tissue inhibitors, proinflammatory cytokines, bone resorption regulators, and heat shock proteins (HSPs) - as potential biomarkers influencing disease susceptibility [9].

SNPs, a common form of DNA sequence variation, play a pivotal role in the etiology of complex diseases [10]. In some cases, infections spread to distant sites and cause severe morbidity or even mortality. Periapical infections and the subsequent inflammation result in bone resorption, driven by osteoclastogenesis through receptor activator of nuclear factor kappa B (RANK) ligand and the accumulation of pro-inflammatory cytokines such as interleukin (IL)-1β, TNF-α, prostaglandin E2, and IL-17 [1].

Several studies have confirmed the pro-inflammatory nature of periapical granulomas, noting higher levels of IL-1β, IL-6, TNF-α, and IL-17 in patients with symptomatic apical periodontitis compared to those with asymptomatic cases [11,12]. Additionally, elevated levels of chemokines such as monocyte chemoattractant protein-1 (MCP-1) and chemokine receptors like CCR2, CCR3, CCR5, and CXCR3 have been observed in periapical lesions, contributing to the recruitment of pro-inflammatory cells to the affected area [1,13].

A study demonstrated that IL-17 receptor antagonist (IL-17RA) knockout (KO) mice experienced greater apical bone loss following pulpal exposure compared to their wild-type (WT) counterparts. This bone loss was accompanied by elevated levels of neutrophils and macrophages and increased bone resorption, highlighting the significant role of IL-17RA signaling in immune modulation during apical periodontitis [14]. Similarly, Sasaki et al. [15] examined the functional effects of Th2-type cytokines such as IL-4 and IL-10 on periapical bone destruction. They found that IL-10 suppresses infection-induced bone loss, while IL-4 showed a different effect [15].

In endodontics, SNPs in various genes significantly impact oral health by influencing infection susceptibility, inflammatory responses, and treatment outcomes. Genes such as MMPs, ILs, bone morphogenetic proteins (BMPs), tumor necrosis factors (TNFs), and signaling molecules like RANK and osteoprotegerin (OPG) have been extensively studied for their role in endodontic diseases. This review aims to synthesize recent findings on the genetic and molecular basis of pulpal and periapical pathologies, with a specific focus on SNPs in these genes. By examining their potential as biomarkers, the review seeks to elucidate their contribution to disease progression, their role in predicting treatment outcomes, and their utility in guiding personalized therapeutic approaches in endodontics.

## Review

Methodology

A comprehensive literature search was conducted across the PubMed, Scopus, and Google Scholar databases to identify studies investigating SNPs associated with dental caries, pulpitis, pulp stones, and periapical periodontitis. The search yielded a total of 120 articles, with 45 articles from PubMed, 38 articles from Scopus, and 37 articles from Google Scholar. After removing duplicates, studies were screened based on predefined inclusion and exclusion criteria.

Studies were included if they provided original data on SNPs in relation to endodontic pathologies. Exclusion criteria were applied to omit unpublished data, non-English articles, and studies limited to abstracts only. A total of 50 articles were excluded, resulting in 70 articles being included in the final review. Additionally, reference lists of relevant studies and recent review articles were examined using computerized searches to ensure the inclusion of the latest research findings. Relevant citations from bibliographies were cross-checked to enhance the comprehensiveness of the review.

Review

MMPs

MMPs are essential for normal tissue remodeling, tissue development, and cell behavior regulation. Proteins from the MMP family play dual roles in the pathophysiology of inflammation by both contributing to tissue damage and activating protective innate and adaptive immune responses. MMPs also have a prominent role in the development of carious lesions, pulpitis, and apical periodontitis [16-18].

MMP1 is typically undetectable in healthy tissues at rest, like many other MMPs. However, fibroblasts, macrophages, endothelial cells, and epithelial cells can produce MMP1 under laboratory conditions. In vivo, its expression is primarily associated with both healthy and pathological tissue remodeling, reflecting its diverse biological functions [19]. MMP1 has been found in dentin, odontoblasts, and saliva, suggesting its involvement in dental caries degradation. Matuszczak et al. [20] found higher salivary concentrations of MMP1 in patients with caries compared to healthy individuals. However, MMP1 levels significantly decreased after oral treatment and cavity sanitation [20]. Trombone et al. [21] examined the relationship between the MMP1 gene -1607 and the risk of periapical lesions; they found that the 1G/2G genotype and the fusion of 1G/2G with 12G/2G significantly increased the risk of periapical lesion development [21].

MMP2, or gelatinase A, is a membrane-bound protein essential for extracellular matrix turnover. It primarily cleaves collagen types IV, V, VII, and XI, as well as gelatin. Specific allele combinations, such as rs243847 with rs2287074 and rs2287074 with rs9923304, have been noted to have significant associations with carious and periradicular lesions [22]. While MMP2 rs2287074 may not directly affect the development of periapical diseases, it could correspond to other genetic regions influencing these conditions [23]. Menezes-Silva et al. [22] studied 16 SNPs in MMP2, MMP3, MMP9, and MMP13; they observed that genetic variations in MMP2 contribute to the progression of dentinal caries and the formation of periapical lesions [22].

MMP3, also known as stromelysin, plays a vital role in extracellular matrix degradation during physiological processes such as embryonic development, reproduction, and tissue remodeling. It is also implicated in several disease processes [24]. Variants such as SNP rs679620 and rs639752 result in the substitution of lysine with glutamine in the protein’s terminal structure and act as markers for increased susceptibility to the rapid progression of dentinal caries and periapical pathologies [1,25].

Elevated levels of MMP8 have been linked to an increased risk of chronic periapical lesions (CPL). The -799 C/T gene polymorphism plays a role in the inflammatory process, making it crucial to identify this genetic variant for targeted treatment in patients at high risk for chronic periapical inflammation [26]. Additionally, mutant alleles of MMP13 have been associated with a risk of dental caries, even after controlling for candidate genes, dentition type, and nutritional factors. This suggests that genetic polymorphisms in MMP13 influence the host’s susceptibility to caries [17].

MMPs are closely associated with the progression of apical periodontitis caused by untreated, deep carious lesions. Furthermore, genetic polymorphisms in MMP and TIMP genes may affect tissue destruction and remodeling at the apex, potentially contributing to the development of inactive apical periodontitis in teeth affected by carious lesions [22].

Table 1 shows the MMPs, SNPs, and their associations.

**Table 1:** Matrix metalloproteinases, single-nucleotide polymorphisms, and their associations MMP1: matrix metalloproteinase 1; MMP2: matrix metalloproteinase 2; MMP3: matrix metalloproteinase 3; MMP8: matrix metalloproteinase 8; MMP13: matrix metalloproteinase 13; SL No.: serial number; SNP: single-nucleotide polymorphism

SL No.	Gene	SNP	Associations
1	MMP1	1607	Primarily associated with both healthy and pathological tissue remodeling, highlighting its diverse biological roles. Increases the chances of periapical lesions [21].
2	MMP2	rs2287074, rs2287074, rs9923304	Impact the advancement of dentinal caries and the development of periapical lesions [23].
3	MMP3	rs679620, rs639752	Act as a marker indicating higher susceptibility to accelerated progression of dentinal caries and periapical pathologies [1,25].
4	MMP8	rs11225395, rs34009635, rs35866072	It is of utmost importance to identify this genetic polymorphism so that targeted treatment can be provided to patients at high risk of suffering from chronic periapical inflammation [26].
5	MMP13	rs2252070	Plays a role in a host’s sensitivity to caries [17].

Tissue Inhibitors of Metalloproteinases (TIMPs)

TIMPs play a crucial role in various dental processes, including dentin formation, the progression of caries, degradation of the hybrid layer, and the development of pulpal and periapical pathologies [22]. While TIMPs are essential for general tissue functions, such as growth and regeneration, they are also involved in pathological processes. Accordingly, tissue samples from periapical lesions typically show elevated levels of TIMPs. Recent studies have revealed an intricate relationship between MMPs and TIMPs in the pathogenesis of periradicular diseases and their subsequent healing [27]. Moreover, after biomechanical preparation and chemical irrigation, studies have observed significant reductions in both MMP and TIMP concentration in patients with periradicular pathologies [28].

Garlet et al. [28] discovered that TIMP1 expression provides a protective counter-response against the degradation of extracellular matrix components and bone resorption. TIMP1 also plays a regulatory role in the progression of periradicular diseases. For instance, higher levels of TIMP1 and TIMP2 are associated with the formation of granulomas and cysts, while TIMP3 and TIMP4 are implicated in the development of chronic apical abscesses [28].

Table 2 shows the TIMPs, SNPs, and their associations.

**Table 2:** Tissue inhibitors of metalloproteinases, single-nucleotide polymorphisms, and their associations SL No.: serial number; SNP: single-nucleotide polymorphism; TIMP1: tissue inhibitor of metalloproteinases 1; TIMP2: tissue inhibitor of metalloproteinases 2; TIMP3: tissue inhibitor of metalloproteinases 3; TIMP4: tissue inhibitor of metalloproteinases 4

SL No.	Gene	SNP	Associations
1	TIMP1	Xp11.3-11.23	Regulates the advancement of periradicular diseases [28].
2	TIMP2	17q25	Associated with a greater number of granulomas and cysts [28].
3	TIMP3	22q12.3	Involved in the development of chronic apical abscesses [28].
4	TIMP4	3p25	Involved in the development of chronic apical abscesses [28].

BMP and Runt-Related Transcription Factor 2 (RUNX-2)

Thuller et al. [29] investigated the relationship between genetic polymorphisms in BMP2 (rs1005464 and rs235768), BMP4 (rs17563), SMAD6 (rs2119261 and rs3934908), and RUNX2 (rs59983488 and rs1200425) with the risk of developing pulp stones. The study found no significant association between polymorphisms in BMP2, BMP4, and RUNX2 and the likelihood of pulp stones. However, polymorphic changes in the SMAD6 gene were linked to an increased risk of pulp stones [29]. Furthermore, the rs2119261 polymorphism in the SMAD6 gene showed a significant association with genotype distribution under the recessive model. Additionally, the T-T haplotype, formed by the rs2119261 and rs3934908 variants, was more prevalent in the comparison group and exhibited a significant correlation with pulp stones [29].

Küchler et al. [30] conducted a study exploring the interactions between SNPs in the BMP2, BMP4, SMAD6, and RUNX2 genes in relation to persistent periradicular diseases. Genomic DNA was analyzed to genotype specific SNPs: rs1005464 and rs235768 in BMP2, rs17563 in BMP4, rs2119261 and rs3934908 in SMAD6, and rs59983488 and rs1200425 in RUNX2 [30]. The study identified a key SNP-SNP interaction model involving rs235768 in BMP2 and rs59983488 in RUNX2 as the most significant factors. This interaction was followed by the combination of rs17563 in BMP4 and rs2119261 in SMAD6. The high-risk genotype was TT + TT in the BMP2-RUNX2 model and GG + TT in the BMP4-SMAD6 model. These interactions between rs235768 in BMP2 and rs59983488 in RUNX2, as well as between rs17563 in BMP4 and rs2119261 in SMAD6, are associated with persistent periradicular periodontitis. Overall, this suggests that the interplay of these SNPs may increase the risk of developing persistent apical periodontitis [30].

Table 3 shows the BMP, SNPs, and their associations.

**Table 3:** Bone morphogenetic proteins, single-nucleotide polymorphisms, and their associations BMP1: bone morphogenetic protein 1; BMP2: bone morphogenetic protein 2; BMP3: bone morphogenetic protein 3; BMP4: bone morphogenetic protein 4; RUNX1: runt-related transcription factor 1; RUNX2: runt-related transcription factor 2; SL No.: serial number; SNP: single-nucleotide polymorphism

SL No.	Gene	SNP	Associations
1	BMP1	8p21.3	Enhances the pulp tissue's natural healing potential [29].
2	BMP2	2q31-33	Promotes the differentiation of human dental pulp cells into odontoblast-like cells [30].
3	BMP3	4q21	Regenerates dentin and pulp [29].
4	BMP4	14q22-q23	Associated with direct pulp capping to increase the formation of reparatory dentin [30].
1	RUNX1	21q22.12	Promotes tooth development [30].
2	RUNX2	6p21.1	Contributes to an increased chance of developing persistent apical periodontitis [30].

Cytokines

TNF-α

TNF-α, a pro-inflammatory cytokine, can influence the outcomes of endodontic therapy. Elevated levels of TNF-α in the dental pulp stimulate pulp cells to produce MMPs [31]. TNF-α is produced by various cells, including macrophages, neutrophils, keratinocytes, fibroblasts, natural killer cells, and T and B lymphocytes [32].

Miron et al. [33] investigated the secretion of TNF-α by macrophages triggered by endodontic pathogens and surface components of bacterial cells. They also aimed to assess the in vitro effects of TNF-α on periostin, cytokine, and MMP secretion, as well as its impact on the viability, proliferation, and mineralization potential of stem cells from the apical papilla (SCAP) [33]. The study found that TNF-α secretion increased when macrophages were stimulated by both Gram-negative and Gram-positive endodontic pathogens. Lipopolysaccharide and lipoteichoic acid also enhanced TNF-α secretion in a dose-dependent manner. Furthermore, TNF-α treatment reduced periostin secretion while increasing the release of various cytokines and MMPs from SCAP [34].

Endodontic pathogens and their surface components stimulate macrophages to release TNF-α, which can negatively affect regenerative endodontic therapy by promoting osteoclastic bone resorption [33]. TNF-α plays a key role in periapical pathogenesis as a potent bone resorption-stimulating mediator [35]. TNF-α also induces the release of multiple pro-inflammatory mediators via SCAP, which intensifies inflammatory reactions within the root canal system and adjacent periradicular tissues. This inflammatory response may impair the differentiation and mineralization capabilities of SCAP, divert their odontogenic differentiation toward osteogenic pathways, and potentially compromise the success of regenerative endodontic therapy [36].

Petean et al. [37] studied the rs1800629 polymorphism in TNF-α and found that it lowers the risk of developing persistent periradicular periodontitis. Endodontic sealers can upregulate inflammatory cytokines, including TNF-α, during the initial stages of inflammation, but they may also downregulate TNF-α and promote tissue regeneration in later stages [38]. The TNF-α polymorphism -308 G/A is associated with a higher susceptibility to apical periodontitis, particularly among heterozygotes (GA) and homozygotes (AA) carrying the variant A allele. Additionally, an inverse relationship was observed between TNF-α levels and the occurrence of symptomatic odontogenic abscesses in patients [39-44].

TNF-α adversely affects SCAP by compromising their viability, proliferation, and mineralization potential. Its ability to promote the secretion of pro-inflammatory cytokines and MMPs worsens inflammatory conditions, leading to tissue degradation and impairing the success of regenerative endodontic therapy [33].

Table 4 shows the TNF-α, SNP, and its associations.

**Table 4:** TNF-α, single-nucleotide polymorphisms, and its associations SNP: single-nucleotide polymorphism; TNF-α: tumor necrosis factor-alpha

Gene	SNP	Associations
TNF-α	6p21	Plays a role in dental pulp healing and reparative dentin formation.
	rs1800629	Affects the outcomes of regenerative endodontic therapy since TNF-α activates osteoclastic bone resorption. TNF-α has a prime involvement in causing periapical pathogenesis as a potent bone resorption-stimulating mediator [35].
	-308 G/A	Lowers the risk of developing persistent periradicular periodontitis [37], and is associated with a higher susceptibility to apical periodontitis [39].

ILs

ILs are essential cytokines that mediate inflammatory responses. Their localized effects include increased leukocyte adherence to endothelial surfaces, activation of lymphocytes, enhanced neutrophil function, promotion of prostaglandin and proteolytic enzyme production, increased bone destruction, and inhibition of bone formation [40].

Cytokines such as IL-1α, IL-1β, IL-6, IL-8, and IL-12p40 play significant roles in the development of apical periodontitis [41-43]. Multiple studies have investigated the polymorphisms of various ILs, including IL-1α, IL-1β, IL-6, TNF-α, IL-8, IL-10, and IL-12, and their association with the occurrence of apical periodontitis [43,44].

The IL-1β polymorphism -511 C/T provides a protective effect in both heterozygous and homozygous individuals. This polymorphism reduces the risk of apical periodontitis, with a twofold decrease in individuals with the CT genotype and nearly a ninefold decrease in those with the TT genotype [44]. An association has been observed between the IL-1β polymorphism and the AA genotype in individuals with deep carious and periradicular lesions. Additionally, the presence of at least one polymorphic T allele in IL-1β increases the risk of persistent apical periodontitis by sevenfold compared to individuals without the polymorphism [39-46].

A study investigated the relationship between the IL-1β gene polymorphism and the failure of endodontic treatments. It revealed a strong connection between recurrent periradicular diseases and the presence of the +3954 C/T polymorphism. This relationship persisted regardless of whether the genotype was heterozygous or homozygous, indicating that the presence of the T allele elevated the risk of recurrent apical periodontitis by seven times compared to individuals without the polymorphism [47,48]. Additionally, genetic variations in the IL-1β gene (rs1143634) are associated with a higher likelihood of external apical root resorption in endodontically treated teeth undergoing orthodontic treatment, especially in individuals homozygous for allele 2 (2/2, TT) [49,50].

A significant relationship was observed between the GG genotype and the G allele of the IL-6 gene polymorphism -174 G/C, particularly in females and individuals aged 35 years or younger who presented with symptomatic odontogenic abscesses [45,46]. Researchers have also examined the polymorphisms of pro-inflammatory IL-8 and anti-inflammatory IL-10 and IL-12. Significant differences were found in the distribution of the A -251 allele of IL-8/CXCL8, particularly among the AA, AT, and wild-type (TT) genotypes. Specifically, the presence of the A allele was linked to chronic non-suppurative apical periodontitis, indicating an increased risk of developing these conditions in both homozygous (AA) and heterozygous (AT) individuals [45,47].

Reports suggest that IL-17 levels are elevated in periodontitis and chronic periradicular lesions [39]. Oseko et al. [49] did a study on mice where the researchers examined the role of IL-17A in periapical lesions; they found that IL-17 plays a crucial role in the formation of these lesions [49]. However, research on IL-17 gene polymorphisms in patients with inflammatory conditions like apical periodontitis remains limited. Most studies have focused on the connection between IL-17 gene polymorphisms and periodontal disease or other immunoinflammatory disorders [39].

Table 5 shows the ILs, SNPs, and their associations.

**Table 5:** Interleukins, single-nucleotide polymorphisms, and their associations IL-1α: interleukin-1 alpha; IL-1β: interleukin-1 beta; IL-6: interleukin-6; IL-8: interleukin-8; IL-12p40: interleukin-12 subunit p40; IL-17: interleukin-17; SL No.: serial number; SNP: single-nucleotide polymorphism

SL No.	Gene	SNP	Associations
1	IL1-α	2 at band q13	Reduction in periapical lesion size [43].
2	IL-1β	2 at the location 113,303,808-113,310,827; -511 C/T; +3954 C/T	Reduces the risk of developing apical periodontitis [50]. Reduces the risk of apical periodontitis [44]. Elevates the risk of recurrent apical periodontitis [48].
3	IL-6	7p21	Contributes to the development of chronic inflammatory lesions [44].
4	IL-8	4q12-q21	Role in inflammation and is associated with endodontic pulpitis [44].
5	IL-12p40	12p40	Role in the development of immune responses and may affect the progression of periapical bone loss [47].
6	IL-17	6p12	Role in the formation of apical lesions [39].

Interferons (IFNs)

IFNs are signaling proteins crucial to both endodontic therapy and immune system function. He et al. [51] discovered that lower concentrations of the inflammatory cytokine IFN-γ stimulate the proliferation and migration of human dental pulp stem cells (DPSCs). Their in vivo results confirmed that IFN-γ plays a regulatory role in the odonto/osteogenic differentiation of DPSCs, along with the signaling pathways involved in this process. This research advances the understanding of IFN-γ’s influence on DPSCs and offers promising therapeutic potential for dentin-pulp tissue engineering in future endodontic treatments [51].

IFN-β promotes bone formation by inhibiting osteoclastogenic activity. In contrast, IFN-gamma (IFN-γ) is produced by activated T-lymphocytes at sites of tissue damage resulting from chronic infections or autoimmune diseases [1]. Pucinelli et al. (2017) studied the expression of IFI16 and IFN-α/β receptors during the initiation and progression of experimental apical periodontitis in mouse teeth. They observed that IFI16 protein expression peaked early after apical periodontitis induction, while the expression of IFN-α/β receptors was highest when the disease was fully established [52].

OPG, RANK, and Receptor Activator of Nuclear Factor Kappa B Ligand (RANKL)

Bone resorption is primarily regulated by the RANK/RANKL/OPG signaling pathway, which plays a central role in bone remodeling [53]. This process is mediated through interactions between RANKL and OPG. RANKL, a soluble mediator belonging to the TNF family, is physiologically expressed but produced in greater amounts during inflammatory processes by T lymphocytes. When RANKL binds to the RANK on bone marrow macrophages, it induces their differentiation into active osteoclasts, which promotes bone resorption [53]. OPG, a soluble decoy receptor produced by osteoblasts in response to anabolic stimuli, competes with RANK for RANKL binding. This inhibits osteoclast formation and suppresses bone resorption [53].

Petean et al. [54] analyzed time as a covariant and found that the genetic polymorphisms RANK rs3826620 and RANKL rs9594738 were associated with a reduced risk of persistent apical periodontitis. Moreover, interactions between TNF-α polymorphism rs1800629 and RANKL polymorphism rs1054016 were linked to a decreased risk of developing persistent apical periodontitis following root canal treatment [54].

In 2022, Petean et al. [37] investigated the imbalance in RANKL and OPG expression in inflammatory conditions, such as periradicular periodontitis. They observed elevated RANKL activity alongside decreased OPG levels, resulting in heightened osteoclastogenic activity and increased bone destruction. Given the multifactorial nature of endodontic failures - which can result from microbial influences, procedural techniques, restorative protocols, and individual host characteristics - it is crucial to examine genetic factors associated with bone remodeling [37]. Thus, genetic polymorphisms in the RANK, RANKL, and OPG genes have significant potential to influence bone remodeling processes and contribute to both disease progression and treatment outcomes.

Nitric oxide (NO) and nitric oxide synthases (NOS)

NO is a gas that serves as a critical signaling molecule within the body. It is synthesized when nitric oxide synthases (NOS) convert L-arginine into L-citrulline, a process essential for various physiological functions [55]. The NOS enzyme exists in three isoforms: neuronal NOS (nNOS), also known as NOS1; inducible NOS (iNOS), also known as NOS2; and endothelial NOS (eNOS), also referred to as NOS3.

Researchers assessed the effects of the genetic polymorphisms NOS2 (rs2297518) and NOS2 (rs2779249) on patients undergoing root canal treatment for asymptomatic periapical lesions. The findings indicated that NOS2 (rs2779249) did not significantly affect the patient’s health. However, NOS2 (rs2297518) was associated with functional impairment, pain, discomfort, and physical and psychological distress [2].

Table 6 shows the NO and NOS, SNPs, and their associations.

**Table 6:** Nitric oxide and nitric oxide synthases, single-nucleotide polymorphisms, and their associations NOS1: nitric oxide synthase 1; NOS2: nitric oxide synthase 2; NOS3: nitric oxide synthase 3; SOCS1: suppressor of cytokine signaling 1; SOCS2: suppressors of cytokine signaling 2; SL No.: serial number; SNP: single-nucleotide polymorphism

SL No.	Gene	SNP	Associations
1	NOS1	rs1879417	Involved in managing dentin hypersensitivity on teeth [2].
2	NOS2	rs2297518 rs2779249	Involved in many physiological and pathological processes [2].
3	NOS3	7q35-7q36	Mediates interactions between inflammation and reparative/regenerative responses in the wounded dental pulp tissue, which is a central determinant of ultimate clinical outcomes [2].
1	SOCS1	rs243327 rs33977706	Role in bone resorption associated with inflammation in apical periodontitis [2].
2	SOCS2		Negatively regulates cytokine signaling and is transcribed in response to cytokine stimulation. It uses several mechanisms to downregulate the signal. Role in resorption associated with inflammation in periodontitis [2].

Suppressors of cytokine signaling (SOCS)

The SOCS consist of eight proteins, known as CIS and SOCS1 through SOCS7. These proteins play a key role in dampening signals from cytokine pathways, which are essential for regulating various important biological processes, such as immune responses and inflammation [56]. Studies have shown that SOCS1, SOCS2, and SOCS3 influence the circulation in patients with periodontitis. This aligns with other research indicating that patients with periodontitis exhibit abnormal expression of immune-related genes in their blood.

An association has been identified between specific polymorphisms in the SOCS1 gene (rs243327 and rs33977706) and patients who have undergone root canal treatment. Consequently, the role of SOCS1 in managing cytokine suppression during inflammatory processes may affect the quality of life of patients receiving root canal treatments [2].

HSPs

HSPs, also known as molecular chaperones, play a crucial role in the synthesis, transport, and proper folding of other proteins. These proteins are essential in the spontaneous immune response by activating macrophages and similar cells. They also participate in the cellular response to lipopolysaccharides, which stimulates the production of inflammatory cytokines [57].

HSP70, in particular, is able to modulate the host's inflammatory immune response. Its involvement in the induction of pro-inflammatory cytokines has been associated with the development of chronic inflammation [58]. In apical periodontitis lesions, the expression of HSP proteins, including DNAJC3, HSPA4, HSPA6, and HSPB1, is increased. Furthermore, SNPs in HSPA1L (rs2075800) and HSPA6 have been linked to the formation of periradicular lesions [59,60].

Wingless-related integration site (WNT genes)

“Single nucleotide polymorphisms (SNPs) in WNT genes” could influence gene and protein function, potentially increasing an individual's susceptibility to periapical lesions. “SNPs in WNT3, WNT3A, WNT5A, WNT8A, WNT9B, and WNT11 genes” were examined to check their association with apical periodontitis. Analysis of single SNP associations showed a trend linking “WNT3 rs9890413 genotypes” under a dominant model, along with “an allelic association for WNT3A rs1745420.” Additionally, haplotypes composed of “WNT3, WNT9B, and WNT3A alleles” were appreciably associated with apical periodontitis [61].

The latest studies indicate that “allele H131 of the FcγRIIA gene,” as well as its combination with “allele NA2 of the FcγRIIIB gene,” may be linked to persistent apical periodontitis [1].

Table 7 shows the HSPs, SNPs, and their associations.

**Table 7:** Heat shock proteins, single-nucleotide polymorphisms, and their associations DNAJC3: DnaJ heat shock protein family (Hsp40) member C3; HSP70: heat shock protein 70; HSPA1L: heat shock protein family A (Hsp70) member 1 like; SL No.: serial number; SNP: single-nucleotide polymorphism; WNT3: wingless-type MMTV integration site family, member 3

SL No.	Gene	SNP	Associations
1	DNAJC3	95,677,139-95,794,988	Role in apical periodontitis [59].
2	HSP70	6p21.33	Helps in tooth development [60].
3	WNT3	rs9890413	Associated with apical periodontitis [61,62].
4	HSPA1L	rs2075800	Formation of periradicular lesions [60].

Clinical applications of SNPs of genes

Gene Therapy

Gene therapy involves the replacement or repair of specific genes to achieve therapeutic benefits [63]. It plays a pivotal role in the success of restorative and endodontic treatments. The dental pulp has long been recognized for its exceptional reparative and regenerative potential, with the ability to differentiate into cells resembling odontoblasts. Experiments have shown that microinjections of growth factor 11 into pulp cells can further enhance their differentiation potential [64].

Genetic Susceptibility and Caries Risk Prediction

Early childhood caries has been associated with several genes and SNPs. The strongest predictors are AMELX_rs17878486, TUFT1_rs2337360, MMP16_rs1042937, and ENAM_rs12640848 [65]. Additionally, SNPs in the AQP5 and ESRRB genes (rs4903419, rs6574293) affect enamel formation, which influences caries susceptibility [66]. Understanding these genetic factors can enable clinicians to promote better oral hygiene, dietary habits, and preventive measures among at-risk patients.

Antimicrobial Therapy

Gene therapy offers the potential to develop targeted antimicrobial treatments for dental diseases. By introducing specific genes into the body, natural defenses can be enhanced to combat particular pathogens, leading to more precise treatments and reducing antibiotic misuse [63].

Future research related to SNPs and genes

Clustered Regularly Interspaced Short Palindromic Repeats (CRISPR)-Cas9 Technology

CRISPR are DNA sequences derived from bacteriophages from previously infected bacteria. This system plays a critical role in bacterial defense by targeting and cleaving invading viral DNA fragments [67]. CRISPR-Cas9 has emerged as a powerful tool for precise genome editing and offers new opportunities in dentistry. It could potentially advance gene therapies, tissue engineering, and regenerative treatments while also targeting harmful microorganisms. As more clinical trials use this technology, it could lead to significant improvements in conservative dentistry and endodontics [68].

Artificial Intelligence (AI)

AI uses algorithms and functions that mimic the human brain [69]. In endodontics, AI has become a valuable tool for diagnostics and personalized treatment planning [70]. AI applications in endodontics include detecting the apical foramen to determine accurate working lengths, distinguishing various peri-radicular diseases, and diagnosing vertical root fractures. In addition, it is also used for evaluating outcomes of regenerative treatments, managing retreatments, and addressing challenges related to complex root morphologies [69].

## Conclusions

Genetic variations in genes related to immune responses and bone repair processes have an influence on how individuals respond to dental treatments. Accordingly, these polymorphisms have the potential to be used as biomarkers to achieve better outcomes in clinical dentistry. Furthermore, understanding the structure of cells, various genetic receptors, and functioning molecules involved in the host's immune-inflammatory response during the progression of acute periodontal disease will upgrade our knowledge of bone biology. This includes the roles of osteoblasts, osteocytes, and osteoclasts in bone turnover, which help identify the host factors that may influence both the progression and healing of lesions. Understanding these genetic factors could help us discover newer therapies by acknowledging the therapeutic targets and developing advanced materials and techniques, eventually improving the outcomes of root canal therapy.

The interactions between the genetic, microbial, and environmental factors are vitally important in the development and treatment of apical periodontitis. By combining insights from genetic research with clinical practices, dental professionals can enhance their management of periapical lesions and achieve improved treatment success rates and better patient outcomes. This comprehensive perspective opens avenues for future research focused on tailoring treatment strategies according to genetic predispositions.
